# Crowded housing, indoor environment and children’s respiratory, allergic and general health in Sweden: a cross-sectional study

**DOI:** 10.1136/bmjopen-2025-106117

**Published:** 2025-09-23

**Authors:** Elodie Eiffener, Rachel Murekatete, Anne-Sophie Merritt, Antonios Georgelis, Catherine Fahlén Zelander, Lina Al-Nahar, Kristina Jakobsson, Maria Albin, Anna Bergström, Marina Jonsson, Charlotta Eriksson

**Affiliations:** 1 Institute of Environmental Medicine, Karolinska Institutet, Stockholm, Sweden; 2 Centre for Occupational and Environmental Medicine, Region Stockholm, Stockholm, Sweden; 3 Division of Occupational and Environmental Medicine, Lund University, Lund, Sweden; 4 School of Public Health and Community Medicine, Institute of Medicine, Sahlgrenska Academy, University of Gothenburg, Gothenburg, Sweden; 5 Department of Clinical Science and Education, Södersjukhuset, Karolinska Institutet, Stockholm, Sweden

**Keywords:** Allergy, Community child health, Cross-Sectional Studies, EPIDEMIOLOGY, Health Equity, PUBLIC HEALTH

## Abstract

**Abstract:**

**Objectives:**

The aim of this study was to analyse associations between crowded housing and children’s indoor living environment, respiratory and allergic disorders and general health.

**Design:**

A cross-sectional study.

**Setting:**

Sweden, using data from the Swedish National Environmental Health Survey 2019.

**Participants:**

The study sample included 48 512 children (aged 6–10 months, 4 years and 12 years). We also investigated associations in vulnerable subgroups, such as children with asthma and those living under unfavourable socioeconomic conditions.

**Primary and secondary outcome measures:**

Primary outcomes in the living environment were at least one sign of mould, poor indoor air quality, unpleasant odours, too warm indoors in summer and too cold indoors in winter. Primary outcomes for children’s health were asthma, airway problems, breathing difficulties, rhinitis symptoms, mould and mites allergy, pollen allergy, furred pet allergy and good general health.

**Results:**

About one in five children lived in an overcrowded home. Factors from the indoor living environment such as perceived poor indoor air quality and mould were significantly associated with crowded housing. Moreover, children who lived in overcrowded conditions were less likely to report good general health than children in non-crowded households (OR 0.64, 95% CI 0.54 to 0.76). This association was even stronger in children with asthma (OR 0.51, 95% CI 0.34 to 0.77). Few significant associations were, however, observed with the respiratory and allergic health outcomes.

**Conclusions:**

Crowded housing is associated both with a poor indoor environment and with poorer general health in children. Children with asthma may experience even poorer general health.

**STRENGTHS AND LIMITATIONS OF THIS STUDY**

- One of the hitherto largest investigations (n=48 512), of the associations between crowded housing, indoor environment and children’s health.
- Population-based study sample including children from all over Sweden in three different age groups, representing infants, young children and school children.
- Findings generalisable to other countries with similar trends in housing and socioeconomic inequalities.
- Cross-sectional design, limiting the possibility to infer causality.
- Potential bias from self-selection into the study (response rate around 40%) and self-reported outcome data which may not always correspond to clinical diagnoses.

## Introduction

 Almost 90% of the Swedish population live in an urban environment.^1^ Urban density prompts unique challenges, not only in urban planning, but also in terms of public health and sustainability.^2^ The urban ecosystem is complex and so are its influences on health.^3^ The heterogeneity of the health burden attributable to environmental exposures between regions, cities and age groups is vast. Moreover, current housing trends, including the lack of affordable homes and crowded housing, tend to reinforce social segregation and escalate disparities in health.^4^

There is no common consensus on how to define crowded housing, and previous research studies have therefore used a variety of different definitions.^5^ In general, however, what is meant is ‘a mismatch between the dwelling and the household’, as broadly defined by the World Health Organisation (WHO) (p22).^6^ One speaks of overcrowding when the number of tenants exceeds the available living space. However, the number of people is not the only criterion, but also the age, sex and relationship of the respective occupants affect the lived experiences of overcrowding. For instance, teenagers seem to be particularly affected by overcrowding, as they have an increased need for privacy.^7^ Further, the concept is not only variously defined, but also differently measured.^6^ The Swedish National Board of Housing, Building and Planning uses three distinct norms to categorise crowded housing, allowing ranges from one to two people per bedroom, whereas partners are always allowed to share a room.^8^ Following European Union (EU) guidelines, children below the age of 12 years can share a bedroom, even up to the age of 18 years, if they are of the same sex.

Already, in the early 19th century, public health practitioners all over Europe and the USA had recognised a link between crowded housing and health.^9^ Consequently, many countries initiated sanitary reforms and the first national measures on overcrowding several decades ago. Yet, nowadays, housing seems to be often overlooked in public health debates.^9^

A recent Swedish review, including studies from all over the world, summarised in what way common characteristics of crowded households may lead to a potentially unhealthy living environment.^5^ The reviewers concluded that crowded housing often coincides with possibly hazardous dwelling conditions, such as the presence of mould, vermin and allergens. In line with these findings, the WHO found that the presence of many tenants in a small living space, with underdimensioned ventilation, may induce increased levels of humidity and mould.^5 6^ This could be due to higher levels of water vapour produced by the human metabolism and household activities, like cooking and showering, often in combination with insufficient ventilation. Lower or higher than optimal indoor temperatures may also contribute to ill-health and are considered as hazardous dwelling conditions, especially in children, as suggested by the WHO.^6^ For instance, cold indoor air is known to be associated with asthma symptoms and indoor heat is likely to cause sleep disturbances.

Children are suggested to be a particularly vulnerable group with respect to environmental exposures, for instance, since their organs, bodily functions and immune system are not fully developed.^4^ However, associations between crowded housing, indoor environment and children’s respiratory and allergic health are complex to study. In Sweden, 14% of 4-year-old children and 9.3% of 12-year-old children have asthma. Asthma prevalence has increased since the ‘50s in Sweden, which is suggested to be due to a combination of both genetic and environmental factors. Asthma is the most common chronic lung disease in children, characterised by irritated and tight airways which make it difficult for children to breathe. The symptoms may vary over time and in intensity. Furthermore, around 24% of 4-year-old and 12-year-old Swedish children have medically diagnosed allergies, with increasing prevalence trends over time. Respiratory allergies, such as hay fever, are very common among young children and can lead to symptoms such as a runny nose, tiredness and reduced quality of life. Unfavourable indoor environments, characterised by, for instance, poor indoor air quality and presence of dust, dampness and mould, have been linked to poorer respiratory health among children, affecting, for example, lung function and lung growth, respiratory symptoms and risk of respiratory morbidity such as asthma.^10 11^ A tentative link between indoor air pollutants and increased asthma incidence and outcome severity has been found in a recent systematic review performed by researchers from all over Europe.^12^ A Swedish study including 3798 children from the BAMSE (Barn, allergi, miljö, Stockholm och epidemiologi) birth cohort indicated that early life exposure to a poor indoor environment is of particular importance and can affect children’s health into adulthood.^11^ Moreover, an American review has shown that overcrowding and subsequent potential lack of privacy may also strain children’s emotional and physical well-being.^13^

Children from socioeconomically disadvantaged areas have previously been found to have a higher prevalence of potentially harmful indoor environmental exposures, also in countries with otherwise high living and economic standards. This was, for instance, reported in a study including 650 participants from 130 families from Malmö, Sweden, analysing the atopic burden of families living in substandard housing.^14^ This population group, characterised by an immigrant background, seemed to be especially affected by both respiratory and allergic symptoms, indicating unmet medical needs. Another study using the same study population but including only children (n=359) highlighted associations between dampness and asthma, as well as mould and headache, respectively.^15^

Overall, however, research on crowded housing in relation to children’s home and health in the current European context is still scarce and difficult to compare due to various definitions and foci.^5^ The main aim of this study was to analyse associations between crowded housing and self-reported state of the indoor living environment, respiratory and allergic disorders and general health of children residing in Sweden. Furthermore, by subgroup analyses in presumed vulnerable groups, we aimed to provide local-level information that could be used for targeted prevention purposes. The following research questions were formulated:

- Is crowded housing associated with an unfavourable indoor living environment, respiratory and allergic disorders and/or poor general health in children?
- Are children in presumably vulnerable subgroups, for example, children with asthma and/or those living in socioeconomically underprivileged areas, more exposed to and affected by overcrowding?

## Methods

### Study population

The study population is based on the Swedish National Environmental Health Survey from 2019 (BMHE19). Every 4 years this survey is sent out by the Swedish Public Health Agency to a random selection of Swedish residents, alternating between children and adults.^16^ The survey design, preparation, administration, as well as population recruitment and data management were performed by and in the responsibility of the Swedish Public Health Agency in collaboration with Statistics Sweden. Each of the 21 counties in Sweden sampled 600 children (200 per age group). Additional densification sampling was done in some geographical areas, as for instance, the metropolitan counties that contain the major cities Stockholm, Malmö and Gothenburg where the biggest vulnerable areas are with low socioeconomic status and overcrowding. The survey collected cross-sectional data on self-reported health, covering questions for instance on asthma, allergies, chronic diseases and general health, as well as potential environmental exposures, including, for example, air quality, noise, green spaces and indoor living environment. The sample population included exclusively children at the ages of 6–10 months, 4 years and 12 years with at least one caretaker residing in Sweden for a minimum of 5 years.^17^ The questionnaire differed slightly for each age group. Most questions were filled out by the caretakers, with some questions being self-reported by the 12-year-old children. The response rate for the survey of the initially 114 591 families contacted was 42.3%, corresponding to 48 512 children (online supplemental table S1), with a partial loss of less than 5% on most survey questions. The survey data were subsequently complemented by the Swedish Public Health Agency with register data, mainly on socioeconomic status. Informed consent was given by the participants by filling in and returning the survey questionnaire. We were provided by the Swedish Public Health Agency with a clean dataset for the analyses of this study.

### Crowded housing

The independent (exposure) variable, crowded housing, in our study was based on two questions from the BMHE19 (‘How many rooms does your home have, excluding the kitchen?’, ‘How many people live in your household?’) and register-based information concerning parental gender and civil status. We assumed each household to have a kitchen and a living room, not used for sleeping. In line with the above-mentioned definition used by the Swedish National Board of Housing, Building and Planning, a household was defined as crowded in our main analyses if there was more than one child per bedroom (norm 3).^8^ We also used a more lenient definition of crowded housing, which allows up to two children per bedroom (norm 2), capturing the even more crowded living, in some of the analyses for sensitivity and comparison purposes. To define vulnerable areas, we used the definition and geographic boundaries provided by the Swedish police.^18^ These areas are often characterised by low socioeconomic status and a high care need index (CNI; a tool for identifying the risk of ill-health based on socioeconomic situation and unfavourable housing conditions).^19^ In addition, we performed stratified analyses based on children with below median household income.

### Indoor living environment and health

The information on the dependent variables (outcomes) originates exclusively from the BMHE19. Not all the outcomes were available for all age groups, as some questions were not applicable in certain age groups, for example, asthma was not assessed in infants. For some variables, we used a combination of different survey questions or dichotomised questions that had more answer options originally in the survey.

For the indoor living environment, we used at least one sign of mould (yes/no), poor indoor air quality (yes/no), unpleasant indoor odours (yes/no), too warm indoors in summer (yes/no) and too cold indoors in winter (yes/no) as outcomes in the analyses.

For the outcomes on child health, we included asthma (yes/no), airway problems (symptoms in nose, throat or trachea) (yes/no), breathing difficulties (yes/no), mould and mites allergy (yes/no), pollen allergy (yes/no), furred pet allergy (yes/no) and good general health (yes/no) as outcomes in the analyses. The definition of asthma was, for instance, based on a combination of three questions from the BMHE19, including self-reported doctors’ diagnosis, asthma medication and prevalence of breathing difficulties in the past 12 months. If at least two of the three questions were answered with yes, we considered the child to have asthma in our analyses, aligning with the definition as used in the national environmental health report, published by the Swedish Public Health Agency.^4^ Among children with asthma, we additionally included the use of asthma medication (yes/no) in our analyses.

### Covariates

The covariates for the main models were defined and selected a priori. Based on the existing literature, we included parental education (elementary school/upper secondary school/university), country of birth (domestic/foreign) and smoking (yes/no); as well as civil status (married/not married) and income (continuous) for each parent respectively as covariates in the analyses. We also included child age (8 months/4 years/12 years), if applicable, and sex (boy/girl) as covariates. Information on the caretakers, except smoking status, originates from registers and the information on the child, along with parental smoking status, stems from the BMHE19.

### Statistical analysis

The data collection and preparation were done by Statistics Sweden (SCB) and the Swedish Public Health Agency. We then performed descriptive analyses to show the baseline characteristics of the study population, including individuals with non-missing information on the exposure and/or outcome variables of interest (n=47 033). Continuous data were shown as medians with IQRs and tested on differences by using the Wilcoxon rank sum test. Categorical data were summarised in frequencies and proportions (n/N) and the Pearson’s χ^2^ test was used for the comparative analysis. Univariate logistic regression was then used to estimate the crude probability of the selected dependent variables on indoor environment and health outcomes in relation to the independent exposure variable crowded housing. Multivariate logistic regression was followed for each dependent variable, respectively, while adjusting for the predefined covariates. We also performed subgroup analyses in children with asthma, children living in vulnerable areas and low-income households, 12-year-old children and children living in the three main metropolitan counties (Stockholm, Västra Götaland and Skåne), respectively. Finally, in a sensitivity analysis, we examined the impact of altering the definition of crowdedness to norm 2. The significance level was set at alpha=0.05. Data are displayed as ORs with 95% c CIs in tables. All statistical analyses in this study were conducted with CRAN R (V.4.3.2). We used Esri’s ArcGIS Pro software (V.3.2.2) for spatial analyses and map creation.

### Patient and public involvement

This study did not include any patients but a random sample of children from the public in Sweden. The public was not involved in the study design or conduct of this study. However, the results and findings from this research will be disseminated to the public in numerous ways. Newsletters will be sent via different channels, including, for instance, the Centre for Occupational and Environmental Medicine, Region Stockholm. Moreover, researchers from the aforementioned Centre have regular visits to and dialogues with professionals’ work in municipalities and healthcare in Stockholm County, where research findings can be discussed directly with the stakeholders involved, aiming to facilitate knowledge translation into policy, from theory to practice. Research findings will also be disseminated through the websites of Karolinska Institute and Region Stockholm.

## Results

### Population characteristics

In total, 9260 out of 47 033 children (19.7%) were defined as living under crowded conditions according to norm 3, compared with 3736 (7.8%) according to norm 2. In the overall sample, around 70% of the households had at least one parent with a university education, had parents of Swedish origin and were living in a house, respectively (table 1). 6% of the households had a smoking parent. Of the children, 11% had asthma and 3% lived in vulnerable areas. Almost half of the study sample were 12-year-old children (43%) while gender was evenly distributed. Comparing children living in crowded versus non-crowded homes, there were several significant differences. For instance, children living under crowded conditions were younger (38% in infants, 23% at 4 years and 12% at 12 years). Furthermore, a higher proportion of children living in crowded conditions had parents of foreign background, lived in an apartment building, had a lower parental education and a lower household income than non-crowded children. Further descriptive statistics on living environment and health outcomes can be found in online supplemental tables S2 and S3.

**Table 1:** Population characteristics, overall and stratified by crowded housing status (norm 3)

Characteristics	N	Overall, N=47 033*†	Crowded housing (norm 3)		P value‡
			No, N=37 773*†	Yes, N=9260*†	
Sex	47 033				0.053
Boy		24 052 (51%)	19 233 (51%)	4819 (52%)	
Girl		22 981 (49%)	18 540 (49%)	4441 (48%)	
Age	47 033				<0.001
8 months		10 885 (23%)	7595 (20%)	3290 (36%)	
4 years		15 693 (33%)	12 149 (32%)	3544 (38%)	
12 years		20 455 (43%)	18 029 (48%)	2426 (26%)	
Parental country of birth§	42 520				<0.001
Domestic		31 799 (75%)	27 802 (81%)	3997 (48%)	
Foreign		10 721 (25%)	6499 (19%)	4222 (51%)	
Housing type	45 571				<0.001
Apartment building		12 949 (28%)	6370 (17%)	6579 (74%)	
House		32 622 (72%)	30 277 (83%)	2345 (26%)	
Parental education level§	46 836				<0.001
Elementary school		884 (1.9%)	307 (0.8%)	577 (6.3%)	
Upper secondary school		13 384 (29%)	10 106 (27%)	3278 (36%)	
University		32 568 (70%)	27 232 (72%)	5336 (58%)	
Smoking parent(s)§	46 826				<0.001
Yes		2776 (5.9%)	1769 (4.7%)	1007 (11%)	
Parent 1 civil status	42 189				<0.001
Married		28 159 (67%)	23 094 (67%)	5065 (65%)	
Not married		14 030 (33%)	11 252 (33%)	2778 (35%)	
Parent 2 civil status	42 378				<0.001
Married		28 242 (67%)	23 127 (67%)	5115 (65%)	
Not married		14 136 (33%)	11 356 (33%)	2780 (35%)	
Parent 1 income	44 294	409 099 (311 041; 549 828)	427 622 (330 600; 574 228)	330 639 (214 708; 437 691)	<0.001
Parent 2 income	43 705	352 697 (254 811; 471 023)	369 682 (278 543; 489 939)	268 572 (123 589; 377 926)	<0.001
Children with asthma	36 069				0.11
Yes		3956 (11%)	3339 (11%)	617 (10%)	
Children living in vulnerable areas	47 033				<0.001
Yes		1418 (3.0%)	430 (1.1%)	988 (11%)	
Children living below median income	36 085				<0.001
Yes		18 043 (50%)	13 514 (45%)	4529 (76%)	
Children in Stockholm County	47 033				<0.001
Yes		14 868 (32%)	10 778 (29%)	4090 (44%)	
Children in Västra Götaland	47 033				<0.001
Yes		3524 (7.5%)	3074 (8.1%)	450 (4.9%)	
Children in Skåne	47 033				0.011
Yes		4708 (10%)	3715 (9.8%)	993 (11%)	
Furred pet ownership	46 858				<0.001
Yes		17 552 (37%)	15 371 (41%)	2181 (24%)	

* n (%); Median (IQR).

† Less than 5% missing for each variable.

‡ Pearson's χ2 test; Wilcoxon rank sum test.

§ Including at least one parent: (a) born in Sweden (domestic); (b) with highest education level; (c) smoking.

The multivariate logistic regressions showed that all outcomes describing a poor indoor living environment were positively associated with crowded housing (table 2). The clearest association was found for poor indoor air quality with an OR of 3.20 (95%CI 2.72 to 3.76). Concerning health, we found an inverse association between living in a crowded home and good general health (OR for good health 0.60, 95% CI 0.51 to 0.72) when compared with children in non-crowded homes. There were also inverse associations with both pollen (OR 0.87, 95% CI 0.77 to 0.98) and furred pet allergies (OR 0.82, 95% CI 0.69 to 0.98). However, none of the other respiratory and allergic health outcomes were significantly associated with crowded housing.

**Table 2:** Results from logistic regression analyses of associations between crowded housing and indicators of poor indoor environment, respiratory and allergic disorders and general health (all children)

Outcome	N cases/exposed*	OR†	95% CI
Indoor living environment			
At least one sign of mould	4002/39 450	1.30	1.20 to 1.42
Poor indoor air quality	756/39 351	3.20	2.72 to 3.76
Unpleasant odour of indoor air	305/16 026	1.69	1.20 to 2.35
Too warm indoors in summer	14 831/39 105	1.40	1.32 to 1.48
Too cold indoors in winter	5908/39 078	1.79	1.67 to 1.92
Health			
Asthma	3281/29 942	0.92	0.82 to 1.03
Airway problems	2140/29 888	0.98	0.86 to 1.12
Breathing difficulties	6947/39 430	0.97	0.90 to 1.04
Rhinitis symptoms	5826/29 452	1.07	0.98 to 1.17
Mould and mites allergy	677/29 339	1.22	0.95 to 1.54
Pollen allergy	4154/29 643	0.87	0.77 to 0.98
Furred pet allergy	1782/29 416	0.82	0.69 to 0.98
Good general health	38 471/39 434	0.60	0.51 to 0.72

* Differences in Ns explained by differing questionnaires per age group, that is, not all questions were answered for all children.

† Multivariate logistic regression models adjusted for parental education, country of birth, civil status, smoking and disposable household income, as well as child age and sex. Unpleasant odour of indoor air was not adjusted for child age as it was only assessed in one age group (12y).

Asthma was reported in 13% of the 4-year-old children and 9.3% of the 12-year-old children. In this subgroup, the home environment outcome variables remained significantly associated with crowded housing, apart from the mould and unpleasant odour variables (online supplemental table S4). The inverse association between crowded housing and general health was strong, with an OR of 0.53 (95% CI 0.36 to 0.81). Otherwise, no other health outcomes were significantly associated with overcrowding. However, we found that children with asthma living in overcrowded conditions used less asthma medication than children with asthma in non-crowded households (OR 0.42, 95% CI 0.20 to 0.94).

**Figure 1:**
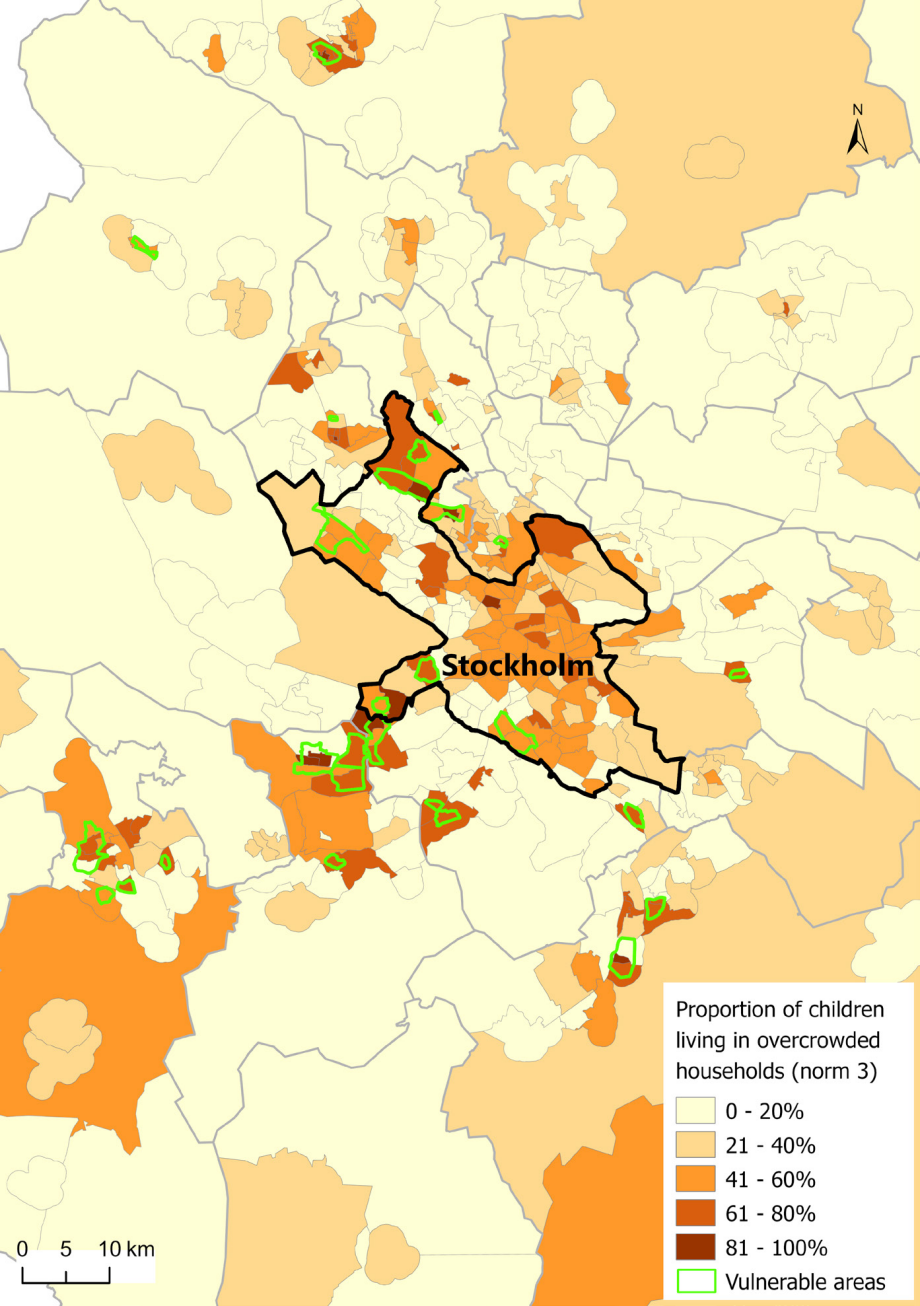
Proportion (%) of children living in overcrowded households (norm 3) within the subsample of respondents to the Swedish National Environmental Health Survey from 2019 in Stockholm County (n=14 868), stratified on regional statistical area level (RegSo). Vulnerable areas overlayed in green.

Concerning children living in vulnerable areas, most risk estimates for the living environment remained elevated but became non-significant due to low power (online supplemental table S5). Similarly, the OR for general health was decreased but non-significant. Figure 1 maps the distribution of children from the BMHE19 living in crowded households with consideration to Stockholm County’s vulnerable areas. Although there is an overlap with these areas, many of the exposed children in our sample live in the central (high-income) areas of Stockholm. Investigating associations in the subgroup of below-median income households did, however, not indicate any strong deviates from the main results (online supplemental table S6).

In the subgroup analyses of children living in the three largest cities Stockholm, Gothenburg and Malmö, respectively, and in the group of 12-year-olds, the trends of the associations could be broadly confirmed, apart from the inverse association with good general health which was statistically significant only in Stockholm County (OR 0.56, 95% CI 0.42 to 0.73) and in the 12-year-olds (OR 0.66, 95% CI 0.52 to 0.84) (online supplemental tables S7–S10). In the subgroup of children in Stockholm County, we additionally found an inverse association between crowded housing and asthma (OR 0.80, 95% CI 0.68 to 0.95) (online supplemental table S7).

**Figure 2:**
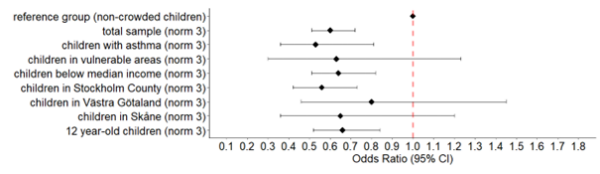
OR and 95% CIs for good general health in different subgroups of children living in crowded households (norm 3) in relation to children living in non-crowded households.

In the sensitivity analysis using norm 2 for crowded housing, the results were somewhat different compared with using norm 3 (online supplemental table S11). The outcomes concerning the indoor living environment remained significantly associated apart from unpleasant odours. Furred pet allergy (OR 0.64, 95% CI 0.44 to 0.90) and good general health (OR 0.75, 95% CI 0.58 to 0.98) prevailed inversely associated; but not pollen allergy. Rhinitis symptoms presented a significant positive association with crowded housing (OR 1.27, 95% CI 1.09 to 1.48), in contrast to breathing difficulties (OR 0.89, 95% CI 0.79 to 1.00)). Figures2  3 also indicate a regional difference with the clearest inverse associations for good general health in Stockholm County compared with the counties of Västra Götaland and Skåne.

**Figure 3:**
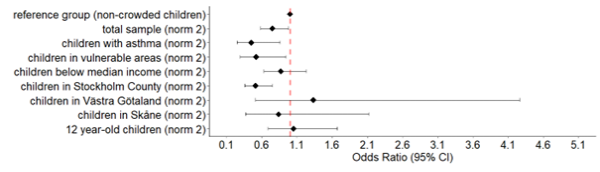
OR and 95% CIs for good general health in different subgroups of children living in crowded households (norm 2) in relation to children living in non-crowded households.

## Discussion

### Summary of findings

Crowded housing, here defined as more than one person per bedroom (except partners), was found among 20% of the children. The prevalence ranged from 12% in 12-year-olds to 38% in infants. The results indicate that crowded housing was associated with both an unfavourable indoor living environment and poorer general health of children up to 12 years old. All factors assessed in the indoor environment were significantly associated with crowded housing, describing inadequate living conditions like poor air quality, mould and unpleasant odours in crowded households. Similarly, children’s general health showed a strong negative association to overcrowding, where children in crowded households reported worse general health than children in non-crowded households. This was even more apparent in children with asthma. The latter seemed also to take less medication for their condition than non-crowded living children with asthma. Pollen and furred pet allergies were, however, less prevalent in children living in crowded conditions. When using a more lenient definition for overcrowding, allowing up to two children per bedroom, most living environment outcomes remained significantly associated with overcrowding. General health remained inversely associated with crowded housing.

### Findings in context

The prevalence of crowded housing in our study (20%) was higher than previous estimates reported for Sweden by the Swedish National Board of Housing, Building and Planning (10% in 2018), but this can likely be explained by differing definitions and assessments.^20^ According to Eurostat, the number of overcrowded homes in Sweden has been rising steadily in the past 10 years, from 12.7% in 2014 to 16.4% in 2023, almost meeting the EU average which contrastingly has been decreasing over the years (16.8% in 2023).^21^ Furthermore, the prevalence of crowded housing seems to be very strongly linked to income in Sweden, more so than in many other European countries.

We found a clear link between overcrowding and poor indoor living environment. This is in concordance with the current literature.^5 22^ Overcrowding, variously defined, may be linked to hazardous dwelling conditions.^5^ While the connection between overcrowding and poor indoor living environment may be apparent, the complexity of epidemiological exposure situations should be highlighted. The attribution of health effects to any exposure is difficult; that is, a detrimental living environment may be associated with crowded housing but also with several other social or behavioural characteristics. In the same way, hazardous dwelling conditions can develop for many reasons other than overcrowding. More in-depth research in this area is needed, including studies using quantitative data and reporting statistical associations, which we have tried to accommodate with our analyses. Also, as Suglia *et al* suggest, associations may be interpreted in two ways.^23^ Either the living conditions serve as a proxy for other factors related to the health outcome or they directly affect health.

Several studies relate the home environment to various paediatric health outcomes, whereas in this study, we mainly found a significant inverse association with general health. In a study from New Zealand, dampness and mould have been shown to present a dose-response relationship with the hospitalisation of children for acute respiratory infections (ARIs).^24^ The authors estimated that 20% of all ARI could be prevented. However, another study from New Zealand found no significant association between mould or dampness in the house and ARI hospitalisation, which is more in line with our findings.^25^ Swedish researchers found a link between residential mould and moisture and recurrent wheezing in children of the BAMSE cohort; a finding that could be confirmed by a recent systematic review.^11 12 26^ Another study from Sweden found rhinitis, asthma and respiratory infections to be related to characteristics of the home environment.^27^ However, some of these studies seem likely to have some residual confounding by socioeconomic status. A study from Finland, analysing associations between housing quality and health in households with children, found associations between general symptoms (eg, headache, fatigue) and thermal discomfort in summer and winter, mould inside and outside the house, as well as unpleasant odours, which is in line with our results; yet the authors remained cautious as to the reliability of the findings.^28^ Clearly, the complexity of epidemiological exposure situations and potential confounding by socioeconomic factors needs to be considered when studying health effects of crowded housing.

According to our findings, children living in crowded households were less likely to have furred pet or pollen allergies than children living in non-crowded households. An unmet higher care need index and poorer access to healthcare may relate to underdiagnosis of allergic diseases in this group compared with the general population and may be one possible explanation.^14 19^ An alternative explanation for the lower risk of pet allergy among crowded living children could be the overall lower rate of furred pet ownership in this group. Yet, Bufford *et al* mention the beneficial effect of dog ownership in early childhood development and atopic diseases, including wheezing.^29^ The findings may also partially be explained by the ‘hygiene hypothesis’, which suggests that early-life exposure to microbes, through overcrowding and/or unhygienic situations, potentially protects from atopic diseases, but the evidence remains hypothetical and inconclusive.^30 31^

In subgroup analyses, we identified children with asthma to have an even poorer general health than children without asthma. Agache *et al* analysed the impact of indoor air pollution, including dampness and mould, on asthma incidence and severity in a recent systematic review.^12^ The findings pointed to a potential link to both outcomes but stressed that the evidence on the impact of indoor pollutants on asthma-related outcomes is relatively uncertain. We also found that crowded children with asthma take less medication compared with non-crowded asthmatic children, thus indicating a higher degree of uncontrolled asthma in this group. In contrast, a cross-sectional study from Sweden, evaluating the association between home environment and asthma medication use, found that people in homes with mouldy odour and dogs would more commonly use asthma medication.^32^ However, the authors did not analyse crowded housing specifically and no other home environment characteristic was related to asthma medication use; the evidence was labelled as having low certainty.^12 32^ The Global Initiative for Asthma described the phenomenon of children not taking their medicine in their strategic report as ‘poor adherence’ and considers this as treatment failure.^33^ They mention inter alia low health literacy, patient concerns and cultural background, cost, misunderstanding and forgetfulness as potential reasons for poor adherence. Also, a burdensome regimen or difficulties in inhaler usage can lead to poor adherence. Patient and caregiver education in children’s asthma management is essential and preferably combined with cultural competence, as language and cultural barriers are seen as potential determinants in treatment adherence.^34 35^ More research is needed to assess this link further, possibly with the use of intervention studies. One may also mention the inverse association found in our study between crowded housing and asthma in children living in Stockholm County. We estimate that this is not an aetiological result but seems rather linked to other factors, such as differences in healthcare access, inappropriate care and underdiagnosing or a result of selection bias.^36 37^

Our subgroup analyses of children living in socioeconomically underprivileged areas (both vulnerable areas and below-median income) did not indicate any strong deviance from the overall patterns of association from the main population. However, one must consider here the potential impact of both differential participation in the study and differential likelihood of diagnosis and care which may have led to an underestimation of the respiratory and allergic disease association in this group. Two studies conducted in a vulnerable area in Malmö, Sweden, have found a link between poor housing standards and high atopic burden in this population, suggesting considerable, unmet medical needs.^14 15^ Thus, identification of population groups experiencing involuntary crowded housing is vital for targeted preventive and health-promotive interventions.^20^

### Limitations and strengths

It should be acknowledged that the study suffers from several limitations. First, it is of cross-sectional design, which limits the possibility to infer causality. Second, since the response rate was rather low (around 40%), there is a risk of differential participation in the study. Third, both the exposure and outcome variables in our analyses stem from self-reported survey data, with answers which may not always correspond to clinical diagnoses. Most likely, this may have resulted in a non-differential misclassification and thus a dilution of any potential associations. Finally, although we took several potentially distorting factors into account, we cannot rule out residual confounding.

The study also inherits several strengths. First, it is one of the hitherto largest investigations of the associations between crowded housing, indoor environment and children’s health. Second, it includes children from all over Sweden in three different age groups, sampled from the general population. Furthermore, using both survey and register data, we were able to adjust the associations for vital sociodemographic determinants such as education, income and parental country of birth; aspects of crucial importance when studying health effects of crowded housing. Third, in comparison to the Swedish general population, we found that our study sample had a similar socioeconomic status, as depicted for instance by comparing median monthly income (Swedish kronor 31 741 vs Swedish kronor 33 330).^38^ Also, the proportion of people with a foreign background in our study population is comparable to the general population.^39^ This supports the generalisability of our findings to the Swedish general population. Finally, we propose that our findings may not only be relevant for the Swedish setting but also for other countries with similar trends in housing and socioeconomic inequalities.

### Practical implications and recommendations

Crowded housing is a multifaceted and complex public health problem and suggestions of political mitigation strategies for crowded housing are outside the scope of this study. However, based on our findings, there are several practical adaptation strategies that could be suggested. For instance, our findings highlight the need for tailored, culturally sensitive interventions, improved asthma education and integrated as well as accessible community healthcare services, in line with conclusions from a recent editorial.^40^ Support systems and decision-support tools that account for socioeconomic and ethnic factors are essential to reduce inequalities among population groups and to improve the health and living environment of young children living in crowded households. For example, we suggest that targeted home visits by trained healthcare professionals could be a potential promising support system for crowded households. Nevertheless, appropriate guidance to housing associations and landlords from the government is also essential to enable adequate living environment and inform respective modification efforts.^22^ Literacy on the associations of housing and health in all stakeholders involved is of key importance.^22^ Clinicians are also encouraged to enquire about housing standards and consider the children’s living environment even more in diagnosis and treatment processes in relation to asthma and respiratory diseases.^22^

## Conclusions

This study indicates that crowded housing in children up to 12 years old is associated with a poor indoor living environment as well as with poorer general health, especially in children with asthma. Few associations were observed regarding the respiratory and allergic outcomes; although, methodological limitations may have influenced our results. Overall, however, the results suggest that it is essential to counteract crowded housing and to provide children with a healthy living environment where they can thrive.

## Supplementary material

10.1136/bmjopen-2025-106117 online supplemental table 1

## Data Availability

Data are available upon reasonable request.

## References

[1] Statistics Sweden (2021). Increasing proportion of people live in Urban areas. https://www.scb.se/en/finding-statistics/statistics-by-subject-area/environment/land-use/localities-and-urban-areas/pong/statistical-news/localities-and-urban-areas-2020/#:~:text=At%20the%20end%20of%20December,more%20than%20100%20000%20inhabitants.

[2] Paköz MZ, Işık M (2022). Rethinking urban density, vitality and healthy environment in the post-pandemic city: The case of Istanbul. Cities.

[3] Dyer GMC, Khomenko S, Adlakha D (2024). Exploring the nexus of urban form, transport, environment and health in large-scale urban studies: A state-of-the-art scoping review. Environ Res.

[4] Folkhälsomyndigheten (2021). Miljöhälsorapport 2021 - Barns miljörelaterade hälsa. https://www.folkhalsomyndigheten.se/publikationer-och-material/publikationsarkiv/m/miljohalsorapport-2021/?pub=88328.

[5] Lorentzen JC, Johanson G, Björk F (2022). Overcrowding and Hazardous Dwelling Condition Characteristics: A Systematic Search and Scoping Review of Relevance for Health. Int J Environ Res Public Health.

[6] World Health Organization (2018). WHO housing and health guidelines. https://iris.who.int/bitstream/handle/10665/276001/9789241550376-eng.pdf?sequence=1.

[7] Lorentzen JC, Georgellis A, Albin M (2024). Residential overcrowding in relation to children’s health, environment and schooling - a qualitative study. Scand J Public Health.

[8] Boverket (2016). Trångboddheten i storstadsregionerna. https://www.boverket.se/globalassets/publikationer/dokument/2016/trangboddheten-i-storstadsregionerna.pdf.

[9] Krieger J, Higgins DL (2002). Housing and health: time again for public health action. Am J Public Health.

[10] Murphy J, Tharumakunarajah R, Holden KA (2023). Impact of indoor environment on children’s pulmonary health. Expert Rev Respir Med.

[11] Thacher JD, Gruzieva O, Pershagen G (2017). Mold and dampness exposure and allergic outcomes from birth to adolescence: data from the BAMSE cohort. *Allergy*.

[12] Agache I, Canelo-Aybar C, Annesi-Maesano I (2024). The impact of indoor pollution on asthma-related outcomes: A systematic review for the EAACI guidelines on environmental science for allergic diseases and asthma. Allergy.

[13] Sandel M, Wright RJ (2006). When home is where the stress is: expanding the dimensions of housing that influence asthma morbidity. Arch Dis Child.

[14] Richter JC, Jakobsson K, Taj T (2018). High burden of atopy in immigrant families in substandard apartments in Sweden - on the contribution of bad housing to poor health in vulnerable populations. World Allergy Organ J.

[15] Oudin A, Richter JC, Taj T (2016). Poor housing conditions in association with child health in a disadvantaged immigrant population: a cross-sectional study in Rosengård, Malmö, Sweden. BMJ Open.

[16] Folkhälsomyndigheten (2023). Public health reporting. https://www.folkhalsomyndigheten.se/the-public-health-agency-of-sweden/public-health-reporting/.

[17] Statistics Sweden (2019). Teknisk rapport BMHE19.

[18] Polisen (2024). Utsatta områden – polisens arbete. https://polisen.se/om-polisen/polisens-arbete/utsatta-omraden/.

[19] Statistics Sweden (2025). Care need index (CNI). https://www.scb.se/vara-tjanster/bestall-data-och-statistik/regionala-statistikprodukter/care-need-index-cni/.

[20] Boverket (2020). Mått på bostadsbristen förslag på hur återkommande bedömningar SKA utföras. https://www.boverket.se/globalassets/publikationer/dokument/2020/matt-pa-bostadsbristen.pdf.

[21] Eurostat (2024). Overcrowding rate by age, sex and poverty status - total population. https://ec.europa.eu/eurostat/databrowser/view/ilc_lvho05a/default/table?lang=en.

[22] Holden KA, Lee AR, Hawcutt DB (2023). The impact of poor housing and indoor air quality on respiratory health in children. Breathe (Sheff).

[23] Franco Suglia S, Duarte CS, Sandel MT (2010). Social and environmental stressors in the home and childhood asthma. J Epidemiol Community Health.

[24] Ingham T, Keall M, Jones B (2019). Damp mouldy housing and early childhood hospital admissions for acute respiratory infection: a case control study. Thorax.

[25] Tin Tin S, Woodward A, Saraf R (2016). Internal living environment and respiratory disease in children: findings from the Growing Up in New Zealand longitudinal child cohort study. Environ Health.

[26] Emenius G, Svartengren M, Korsgaard J (2004). Indoor exposures and recurrent wheezing in infants: a study in the BAMSE cohort. Acta Paediatr.

[27] Wang J, Engvall K, Smedje G (2014). Rhinitis, Asthma and Respiratory Infections among Adults in Relation to the Home Environment in Multi-Family Buildings in Sweden. PLoS ONE.

[28] Turunen M, Iso-Markku K, Pekkonen M (2017). Statistical associations between housing quality and health among Finnish households with children - Results from two (repeated) national surveys. Sci Total Environ.

[29] Bufford JD, Reardon CL, Li Z (2008). Effects of dog ownership in early childhood on immune development and atopic diseases. Clin Exp Allergy.

[30] Casas L, Tischer C, Täubel M (2016). Pediatric Asthma and the Indoor Microbial Environment. *Curr Environ Health Rep*.

[31] Matricardi PM, Ronchetti R (2001). Are infections protecting from atopy?. http://journals.lww.com/co-allergy.

[32] Wang J, Engvall K, Smedje G (2017). Exacerbation of asthma among adults in relation to the home environment in multi-family buildings in Sweden. Int J Tuberc Lung Dis.

[33] Global Initiative for Asthma (2024). Global strategy for asthma management and prevention. https://ginasthma.org/wp-content/uploads/2024/05/GINA-2024-Strategy-Report-24_05_22_WMS.pdf.

[34] Shipp CL, Gergen PJ, Gern JE (2023). Asthma Management in Children. J Allergy Clin Immunol Pract.

[35] McQuaid EL (2018). Barriers to medication adherence in asthma: The importance of culture and context. Ann Allergy Asthma Immunol.

[36] Jonsson M, Egmar A-C, Kiessling A (2012). Adherence to national guidelines for children with asthma at primary health centres in Sweden: potential for improvement. Prim Care Respir J.

[37] Ingemansson M, Wettermark B, Jonsson EW (2012). Adherence to guidelines for drug treatment of asthma in children: potential for improvement in Swedish primary care. *Qual Prim Care*.

[38] Statistics Sweden (2023). Average monthly salary by occupation, 2023. https://www.scb.se/en/finding-statistics/statistics-by-subject-area/labour-market/wages-salaries-and-labour-costs/wage-and-salary-structures-and-employment-in-the-municipalities/pong/tables-and-graphs/average-monthly-salary-by-occupation/.

[39] Statistics Sweden (2023). Sweden’s population in summary 1960-2023. https://www.scb.se/en/finding-statistics/statistics-by-subject-area/population/population-composition/population-statistics/pong/tables-and-graphs/population-statistics---summary/swedens-population-in-summary-1960-2023/.

[40] Nordlund B (2024). Inequalities in care and the burden of wheeze and asthma in young children from diverse socioeconomic and ethnic backgrounds. Thorax.

