# Supplementary material for: Crowded housing, indoor environment and children’s respiratory, allergic and general health in Sweden: a cross-sectional study
Source: BMJ Open. 2025 Sep 23;15(9):e106117. doi: 10.1136/bmjopen-2025-106117 (PMC12458785; doi:10.1136/bmjopen-2025-106117)
Supplement: online supplemental table 1 [file bmjopen-15-9-s001.docx]

**Supplemental material**

**Table S1**. Description of the sample and response rate, age-specific and total.

|  | **Age** | | | |
| --- | --- | --- | --- | --- |
|  | *6-10 months* | *4 years* | *12 years* | *Total* |
| **Target population (N)** | 38,502 | 113,445 | 113,431 | 265,378 |
| **Sample (N)** | 23,516 | 37,249 | 53,826 | 114,591 |
| **Responders (N)** | 11,065 | 16,072 | 21,375 | 48,512 |
| **Response rate (%)** | 47.1 | 43.1 | 39.7 | 42.3 |

**Table S2.** Descriptive statistics on crowded housing and indoor living environment (all children).

|  |  |  | **Crowded Housing Norm 3** | |  |
| --- | --- | --- | --- | --- | --- |
| **Characteristics** | **N** | **Overall**,  N = 47,033 ^a^ | **No**, N = 37,773 ^a^ | **Yes**, N = 9,260 ^a^ | **p-value** ^b^ |
| **At least one sign of mold** | 46,873 |  |  |  | <0.001 |
| Yes |  | 4,896 (10%) | 3,634 (9.7%) | 1,262 (14%) |  |
| **Poor indoor air quality** | 46,746 |  |  |  | <0.001 |
| Yes |  | 1,049 (2.2%) | 511 (1.4%) | 538 (5.9%) |  |
| **Unpleasant odor of indoor air** | 20,275 |  |  |  | <0.001 |
| Yes |  | 453 (2.2%) | 345 (1.9%) | 108 (4.6%) |  |
| **Too warm indoors in summer** | 46,358 |  |  |  | <0.001 |
| Yes |  | 17,573 (38%) | 13,381 (36%) | 4,192 (47%) |  |
| **Too cold indoors in winter** | 46,337 |  |  |  | <0.001 |
| Yes |  | 7,509 (16%) | 5,012 (13%) | 2,497 (28%) |  |
| ^a^ n (%); Median (IQR) | | | | | |
| ^b^ Pearson's Chi-squared test; Wilcoxon rank sum test | | | | | |

**Table S3.** Descriptive statistics on crowded housing and health outcomes (all children).

|  |  |  | **Crowded Housing Norm 3** | | |
| --- | --- | --- | --- | --- | --- |
| **Characteristics** | **N** | **Overall,**  N = 47,033 ^a^ | **No**, N = 37,773 ^a^ | **Yes**, N = 9,260 ^a^ | **p-value** ^b^ |
| **Asthma** | 36,069 |  |  |  | 0.11 |
| Yes |  | 3,956 (11%) | 3,339 (11%) | 617 (10%) |  |
| **Airway problems** | 35,988 |  |  |  | <0.001 |
| Yes |  | 2,648 (7.4%) | 2,124 (7.1) | 524 (8.9%) |  |
| **Breathing difficulties** | 46,849 |  |  |  | 0.4 |
| Yes |  | 8,114 (17%) | 6,490 (17%) | 1,624 (18%) |  |
| **Rhinitis symptoms** | 35,467 |  |  |  | 0.8 |
| Yes |  | 7,319 (21%) | 6,114 (21%) | 1,205 (21%) |  |
| **Mold and mites allergy** | 35,287 |  |  |  | 0.017 |
| Yes |  | 879 (2.5%) | 711 (2.4%) | 168 (2.9%) |  |
| **Pollen allergy** | 35,662 |  |  |  | <0.001 |
| Yes |  | 5,234(15%) | 4,583 (15%) | 651 (11%) |  |
| **Furred pet allergy** | 35,379 |  |  |  | <0.001 |
| Yes |  | 2,262 (6.4%) | 1,974 (6.7%) | 288 (5.0%) |  |
| **Good general health** | 46,845 |  |  |  | <0.001 |
| Yes |  | 45,458 (97%) | 36,650 (97%) | 8,808 (96%) |  |
| ^a^ n (%); Median (IQR) | | | | | |
| ^b^ Pearson's Chi-squared test; Wilcoxon rank sum test | | | | | |

**Table S4**. Results from logistic regression analyses of associations between crowded housing and indicators of poor indoor environment, respiratory and allergic disorders, and general health (children with asthma).

| **Outcome** | **N Cases/Exposed** ^a^ | **OR** ^b^ | **95% CI** ^c^ |
| --- | --- | --- | --- |
| **Indoor living environment** |  |  |  |
| At least one sign of mold | 382/3,270 | 1.35 | 0.99-1.82 |
| Poor indoor air quality | 96/3,265 | 3.89 | 2.45-6.15 |
| Unpleasant odor of indoor air | 41/1,474 | 1.87 | 0.70-4.48 |
| Too warm indoors in summer | 1,308/3,254 | 1.51 | 1.22-1.87 |
| Too cold indoors in winter | 515/3,253 | 2.06 | 1.60-2.64 |
| **Health** |  |  |  |
| Airway problems | 832/3,272 | 0.96 | 0.75-1.21 |
| Breathing difficulties | 2,744/3,277 | 1.08 | 0.80-1.49 |
| Rhinitis symptoms | 1,335/3,214 | 1.25 | 0.99-1.57 |
| Mold and mites allergy | 278/3,153 | 1.41 | 0.91-2.14 |
| Pollen allergy | 1,006/3,216 | 1.10 | 0.83-1.46 |
| Furred pet allergy | 680/3,178 | 0.78 | 0.56-1.07 |
| Good general health | 3,087/3,271 | 0.53 | 0.36-0.81 |
| Use of asthma medication | 3,238/3,281 | 0.42 | 0.20-0.94 |
| ^a^ Differences in Ns explained by differing questionnaires per age group, i.e. not all questions were answered by all children. ^b^ OR = Odds ratio. Multivariate logistic regression models adjusted for parental education, country of birth, civil status, smoking and disposable household income, as well as child age and sex. Unpleasant odor of indoor air was not adjusted for child age as it was only assessed in one age group (12y).  ^c^ CI = Confidence interval | | | |

**Table S5**. Results from logistic regression analyses of associations between crowded housing and indicators of poor indoor environment, respiratory and allergic disorders, and general health (children in vulnerable areas).

| **Outcome** | **N Cases/Exposed** ^a^ | **OR** ^b^ | **95% CI** ^c^ |
| --- | --- | --- | --- |
| **Indoor living environment** |  |  |  |
| At least one sign of mold | 162/980 | 1.12 | 0.75-1.71 |
| Poor indoor air quality | 63/976 | 1.48 | 0.79-2.92 |
| Unpleasant odor of indoor air | 19/348 | 1.04 | 0.35-3.45 |
| Too warm indoors in summer | 374/916 | 1.12 | 0.81-1.55 |
| Too cold indoors in winter | 327/930 | 1.26 | 0.91-1.76 |
| **Health** |  |  |  |
| Asthma | 68/739 | 0.93 | 0.51-1.72 |
| Airway problems | 73/730 | 1.03 | 0.59-1.87 |
| Breathing difficulties | 133/983 | 1.36 | 0.87-2.18 |
| Rhinitis symptoms | 202/719 | 0.76 | 0.52-1.13 |
| Mold and mites allergy | 29/690 | 1.46 | 0.60-3.95 |
| Pollen allergy | 97/712 | 0.65 | 0.39-1.08 |
| Furred pet allergy | 22/691 | 0.51 | 0.19-1.37 |
| Good general health | 923/981 | 0.63 | 0.30-1.23 |
| ^a^ Differences in Ns explained by differing questionnaires per age group, i.e. not all questions were answered by all children. ^b^ OR = Odds ratio. Multivariate logistic regression models adjusted for parental education, country of birth, civil status, smoking and disposable household income, as well as child age and sex. Unpleasant odor of indoor air was not adjusted for child age as it was only assessed in one age group (12y).  ^c^ CI = Confidence interval | | | |

**Table S6**. Results from logistic regression analyses of associations between crowded housing and indicators of poor indoor environment, respiratory and allergic disorders, and general health (children from below-median income households).

| **Outcome** | **N Cases/Exposed** ^a^ | **OR** ^b^ | **95% CI** ^c^ |
| --- | --- | --- | --- |
| **Indoor living environment** |  |  |  |
| At least one sign of mold | 1,512/13,325 | 1.35 | 1.18-1.54 |
| Poor indoor air quality | 350/13,287 | 2.72 | 2.13-3.46 |
| Unpleasant odor of indoor air | 117/5,315 | 1.89 | 1.19-2.97 |
| Too warm indoors in summer | 4,965/13,116 | 1.49 | 1.36-1.63 |
| Too cold indoors in winter | 2,419/13,122 | 1.74 | 1.56-1.94 |
| **Health** |  |  |  |
| Asthma | 1,490/13,333 | 0.92 | 0.79-1.06 |
| Airway problems | 1,074/13,294 | 0.96 | 0.81-1.13 |
| Breathing difficulties | 2,593/13,306 | 0.86 | 0.76-0.96 |
| Rhinitis symptoms | 2,460/13,084 | 1.03 | 0.91-1.15 |
| Mold and mites allergy | 281/13,027 | 1.16 | 0.84-1.57 |
| Pollen allergy | 1,493/13,161 | 0.92 | 0.78-1.07 |
| Furred pet allergy | 685/13,059 | 0.81 | 0.64-1.01 |
| Good general health | 12,862/13,312 | 0.64 | 0.51-0.82 |
| ^a^ Differences in Ns explained by differing questionnaires per age group, i.e. not all questions were answered by all children. ^b^ OR = Odds ratio. Multivariate logistic regression models adjusted for parental education, country of birth, civil status, smoking and disposable household income, as well as child age and sex. Unpleasant odor of indoor air was not adjusted for child age as it was only assessed in one age group (12y).  ^c^ CI = Confidence interval | | | |

**Table S7.** Results from logistic regression analyses of associations between crowded housing and indicators of poor indoor environment, respiratory and allergic disorders, and general health (children in Stockholm County).

| **Outcome** | **N Cases/Exposed** ^a^ | **OR** ^b^ | **95% CI** ^c^ |
| --- | --- | --- | --- |
| **Indoor living environment** |  |  |  |
| At least one sign of mold | 1,194/12,434 | 1.27 | 1.10-1.46 |
| Poor indoor air quality | 318/12,399 | 2.66 | 2.08-3.41 |
| Unpleasant odor of indoor air | 108/4,089 | 1.34 | 0.76-2.25 |
| Too warm indoors in summer | 5,121/12,322 | 1.23 | 1.13-1.35 |
| Too cold indoors in winter | 2,242/12,317 | 1.50 | 1.35-1.66 |
| **Health** |  |  |  |
| Asthma | 1,151/9,063 | 0.80 | 0.68-0.95 |
| Airway problems | 732/9,040 | 0.91 | 0.74-1.10 |
| Breathing difficulties | 2,344/12,418 | 0.96 | 0.86-1.07 |
| Rhinitis symptoms | 1,687/8,904 | 1.05 | 0.91-1.21 |
| Mold and mites allergy | 146/8,860 | 1.08 | 0.68-1.67 |
| Pollen allergy | 1,116/8,965 | 0.76 | 0.62-0.92 |
| Furred pet allergy | 447/8,885 | 0.67 | 0.49-0.91 |
| Good general health | 12,120/12,422 | 0.56 | 0.42-0.73 |
| ^a^ Differences in Ns explained by differing questionnaires per age group, i.e. not all questions were answered by all children. ^b^ OR = Odds ratio. Multivariate logistic regression models adjusted for parental education, country of birth, civil status, smoking and disposable household income, as well as child age and sex. Unpleasant odor of indoor air was not adjusted for child age as it was only assessed in one age group (12y).  ^c^ CI = Confidence interval | | | |

**Table S8**. Results from logistic regression analyses of associations between crowded housing and indicators of poor indoor environment, respiratory and allergic disorders, and general health (children living in Västra Götaland).

| **Outcome** | **N Cases/Exposed** ^a^ | **OR** ^b^ | **95% CI** ^c^ |
| --- | --- | --- | --- |
| **Indoor living environment** |  |  |  |
| At least one sign of mold | 304/2,790 | 1.74 | 1.16-2.51 |
| Poor indoor air quality | 43/2,782 | 4.79 | 2.25-10.0 |
| Unpleasant odor of indoor air | 59/2,622 | 2.09 | 0.94-4.33 |
| Too warm indoors in summer | 751/2,763 | 1.56 | 1.16-2.08 |
| Too cold indoors in winter | 328/2,766 | 1.84 | 1.30-2.60 |
| **Health** |  |  |  |
| Asthma | 213/2,721 | 0.86 | 0.48-1.46 |
| Airway problems | 170/2,715 | 0.77 | 0.41-1.36 |
| Breathing difficulties | 294/2,797 | 0.91 | 0.57-1.41 |
| Rhinitis symptoms | 743/2,676 | 0.80 | 0.58-1.11 |
| Mold and mites allergy | 94/2,651 | 0.89 | 0.40-1.80 |
| Pollen allergy | 618/2,694 | 0.75 | 0.52-1.06 |
| Furred pet allergy | 227/2,657 | 0.61 | 0.32-1.06 |
| Good general health | 2,677/2,798 | 0.80 | 0.46-1.45 |
| ^a^ Differences in Ns explained by differing questionnaires per age group, i.e. not all questions were answered by all children. ^b^ OR = Odds ratio. Multivariate logistic regression models adjusted for parental education, country of birth, civil status, smoking and disposable household income, as well as child age and sex. Unpleasant odour of indoor air was not adjusted for child age as it was only assessed in one age group (12y).  ^c^ CI = Confidence interval | | | |

**Table S9**. Results from logistic regression analyses of associations between crowded housing and indicators of poor indoor environment, respiratory and allergic disorders, and general health (children living in Skåne).

| **Outcome** | **N Cases/Exposed** ^a^ | **OR** ^b^ | **95% CI** ^c^ |
| --- | --- | --- | --- |
| **Indoor living environment** |  |  |  |
| At least one sign of mold | 443/4,029 | 1.31 | 1.02-1.69 |
| Poor indoor air quality | 76/4,020 | 3.14 | 1.86-5.26 |
| Unpleasant odor of indoor air | 18/1,140 | 3.74 | 0.96-12.8 |
| Too warm indoors in summer | 1,524/3,990 | 1.70 | 1.43-2.03 |
| Too cold indoors in winter | 636/3,988 | 1.56 | 1.25-1.93 |
| **Health** |  |  |  |
| Asthma | 260/2,561 | 1.01 | 0.68-1.47 |
| Airway problems | 187/2,560 | 0.98 | 0.63-1.50 |
| Breathing difficulties | 697/4,023 | 0.82 | 0.65-1.04 |
| Rhinitis symptoms | 411/2,531 | 1.30 | 0.93-1.78 |
| Mold and mites allergy | 79/2,523 | 1.03 | 0.44-2.20 |
| Pollen allergy | 272/2,531 | 0.83 | 0.51-1.31 |
| Furred pet allergy | 115/2,527 | 0.87 | 0.42-1.65 |
| Good general health | 3,950/4,025 | 0.65 | 0.36-1.20 |
| ^a^ Differences in Ns explained by differing questionnaires per age group, i.e. not all questions were answered by all children. ^b^ OR = Odds ratio. Multivariate logistic regression models adjusted for parental education, country of birth, civil status, smoking and disposable household income, as well as child age and sex. Unpleasant odour of indoor air was not adjusted for child age as it was only assessed in one age group (12y).  ^c^ CI = Confidence interval | | | |

**Table S10**. Results from logistic regression analyses of associations between crowded housing and indicators of poor indoor environment, respiratory and allergic disorders, and general health (12-year-old children).

| **Outcome** | **N  Cases/Exposed** ^a^ | **OR** ^b^ | **95% CI** ^c^ |
| --- | --- | --- | --- |
| **Indoor living environment** |  |  |  |
| At least one sign of mold | 1,487/16,056 | 1.70 | 1.43-2.00 |
| Poor indoor air quality | 195/16,018 | 3.92 | 2.75-5.54 |
| Unpleasant odor of indoor air | 305/16,026 | 1.69 | 1.20-2.35 |
| Too warm indoors in summer | 4,818/15,927 | 1.45 | 1.28-1.63 |
| Too cold indoors in winter | 1,754/15,921 | 1.99 | 1.71-2.31 |
| **Health** |  |  |  |
| Asthma | 1,488/16,122 | 0.93 | 0.75-1.14 |
| Airway problems | 973/16,099 | 1.11 | 0.88-1.39 |
| Breathing difficulties | 1,805/16,091 | 1.03 | 0.85-1.23 |
| Rhinitis symptoms | 4,210/15,894 | 1.00 | 0.88-1.14 |
| Mold and mites allergy | 586/15,728 | 1.29 | 0.98-1.68 |
| Pollen allergy | 3,555/15,974 | 0.88 | 0.76-1.01 |
| Furred pet allergy | 1,453/15,784 | 0.88 | 0.71-1.08 |
| Good general health | 15,415/16,094 | 0.66 | 0.52-0.84 |
| ^a^ Differences in Ns explained by differing questionnaires per age group, i.e. not all questions were answered by all children. ^b^ OR = Odds ratio. Multivariate logistic regression models adjusted for parental education, country of birth, civil status, smoking and disposable household income, as well as child sex.  ^c^ CI = Confidence interval | | | |

**Table S11.** Results from logistic regression analyses of associations between crowded housing and indicators of poor indoor environment, respiratory and allergic disorders, and general health (all children, norm 2 of crowded housing).

| **Outcome** | **N Cases/Exposed** ^a^ | **OR** ^b^ | **95% CI** ^c^ |
| --- | --- | --- | --- |
| **Indoor living environment** |  |  |  |
| At least one sign of mold | 4,002/39,450 | 1.19 | 1.05-1.35 |
| Poor indoor air quality | 756/39,351 | 2.61 | 2.13-3.20 |
| Unpleasant odor of indoor air | 305/16,026 | 1.66 | 0.91-2.83 |
| Too warm indoors in summer | 14,831/39,105 | 1.30 | 1.19-1.42 |
| Too cold indoors in winter | 5,908/39,078 | 1.87 | 1.69-2.06 |
| **Health** |  |  |  |
| Asthma | 3,281/29,942 | 0.95 | 0.77-1.15 |
| Airway problems | 2,140/29,888 | 0.98 | 0.78-1.21 |
| Breathing difficulties | 6,947/39,430 | 0.89 | 0.79-1.00 |
| Rhinitis symptoms | 5,826/29,452 | 1.27 | 1.09-1.48 |
| Mold and mites allergy | 677/29,339 | 1.34 | 0.89-1.96 |
| Pollen allergy | 4,154/29,643 | 0.90 | 0.72-1.11 |
| Furred pet allergy | 1,782/29,416 | 0.64 | 0.44-0.90 |
| Good general health | 38,471/39,434 | 0.75 | 0.58-0.98 |
| ^a^ Differences in Ns explained by differing questionnaires per age group, i.e. not all questions were answered by all children. ^b^ OR = Odds ratio. Multivariate logistic regression models adjusted for parental education, country of birth, civil status, smoking and disposable household income, as well as child age and sex. Unpleasant odour of indoor air was not adjusted for child age as it was only assessed in one age group (12y).  ^c^ CI = Confidence interval | | | |
