# Supplementary material for: Crowded housing, indoor environment and children’s respiratory, allergic and general health in Sweden: a cross-sectional study
Source: BMJ Open. 2025 Sep 23;15(9):e106117. doi: 10.1136/bmjopen-2025-106117 (PMC12458785; doi:10.1136/bmjopen-2025-106117)
Supplement: Supplementary file 2 [file bmjopen-2025-106117.reviewer_comments.pdf]

PEER REVIEW HISTORY

BMJ Open publishes all reviews undertaken for accepted manuscripts. Reviewers are asked to complete a checklist review form and are provided with free text boxes to elaborate on their assessment. These free text comments are reproduced below.

ARTICLE DETAILS

Title (Provisional)

Crowded housing, indoor environment and children's respiratory, allergic and general health in Sweden: a cross-sectional study

Authors

Eiffener, Elodie; Murekatete, Rachel Mundeli Mundeli; Merritt, Anne-Sophie; Georgelis, Antonios; Fahlén Zelander, Catherine; Al-Nahar, Lina; Jakobsson, Kristina; Albin, Maria; Bergstrom, Anna; Jonsson, M.; Eriksson, Charlotta

---

VERSION 1 - REVIEW

**Reviewer**	**1**
**Name**	**Chandrasekaran, Venkatesh**
**Affiliation**	**Jawaharlal Institute of Postgraduate Medical Education and Research, Department of Pediatrics**
**Date**	**11-Aug-2025**
**COI**	**None**

An interesting study involving a large number of children to assess whether overcrowding is associated with unfavorable indoor living environment, respiratory and allergic disorders and poor general health. This study also attempts to assess whether children with asthma or those from economically underprivileged areas are more exposed or affected by overcrowding.

The outcome measures have been collected as yes/no answers from the respondents making the measure very subjective

The authors have used two different sets of criteria to measure overcrowding- a stringent criterion (norm 3) in which if more than one person sharing the same bedroom other than their partner and a liberal criterion (norm 2) in which >2 individuals sharing a bedroom is considered overcrowding. In this context, it would have been interesting if the authors had also collected information about the size of the bedroom, as well as cultural context facilitating sharing of the same space. For instance, it is common among Indian Children to share bed with their parents in most households until they become adolescents.

Interestingly the authors have used a term “criterium” which I understand it to be criterion.

No formal sample size calculation has been mentioned for this study. The study has reportedly used multi-cluster sampling to recruit children in the study with each county recruiting about 600 children with 200 in each of the 3 age group categories, 6-10 months, 4 years and 12 years. It is not clear how the rest of the sample was recruited considering there were only three counties mentioned in the results- Stockholm, Vastra Gotaland and Skane. It is also not clear what was the rationale for selecting these different age groups.

The authors considered a diagnosis of asthma in the child if the parents have been told by the doctors about it, or if the child is on asthma medications or prevalence of breathing difficulty in the past 12 months. In this context, it is important to note that some of the children would have outgrown the symptoms, might not be on medications currently and might not be having any symptoms in the past 12 months making the measurement inaccurate.

Parental perception of good general health of their children might vary considerably. Instead, the authors could have used a surrogate like the absence of poor weight gain and or poor appetite, frequent infections or need for frequent hospitalizations due to ill health.

Similarly parental perception of poor air quality might also not be reliable. Instead, the authors could have used air quality index values of the neighborhoods acquired from nearest weather monitoring stations.

Similarly, perception of too much cold indoors in the winter and too much heat in the summers could be very subjective

A STROBE compliant study flow diagram depicting patient flow into the study is missing and the authors have stated it is not applicable in the STROBE checklist, whereas it is very much needed to understand how many were found to be eligible for participation, how many met the study inclusion criteria, how many were excluded and for what reasons and how many were analysed finally.

In table 1 the number of children belonging to different counties do not count up to 47,033.

Reference number 8 appears to be incomplete.

Reviewer 2

Name Chen, Ren

Affiliation Anhui Medical University, Department of Health Service Administration

Date 12-Aug-2025

COI None

Thank you for the invitation to review this manuscript. This article examines the relationship

between indoor living environments, crowded housing, and childhood allergies and respiratory diseases in Sweden, providing insights for other scholars studying the environment and children's health. I have some concerns about certain parts of the manuscript and hope that the author will address these issues after careful consideration.

1. In this study, the health outcomes for children included asthma, airway issues, breathing difficulties, mold and dust mite allergies, pollen allergies, furry pet allergies, and overall good health. Furthermore, the entire study primarily focused on children's allergies and respiratory diseases. However, the current title is "......and children's health......," which may not align closely with the theme and feels somewhat broad. It is recommended that the author consider revising the title to make it more rigorous and targeted.

2. In the background section, the author describes the impact of overcrowded housing and poor indoor environments on children's health, as well as previous research findings. However, there is limited discussion of children's allergies and respiratory diseases themselves. Even when mentioned, they are often discussed in conjunction with poor living conditions. Therefore, it is recommended that this aspect be further elaborated upon.

3. Adding information about the current situation of respiratory diseases and allergies among Swedish children to the research background would make the article more complete.

4. The review of previous studies could be more detailed to present a clearer picture for the readers. For example, "A study of XX participants (here you can clearly state what the sample is) using XX method found XX."

5. In the subgroup analysis of the statistical analysis, what is your definition of the three major metropolitan counties of Stockholm, Västra Götalands, and Skåne? Or what is the basis for your selection? Please provide additional information to clarify this point.

6. It is recommended that the description of population characteristics be more detailed.

7. When describing the results, subjective inferences should not be made. The results section should only objectively present your findings.

8. Specific, actionable recommendations should be proposed in response to the identified issues and challenges.

9. Carefully check the correspondence between text descriptions, charts, and textual data.

VERSION 1 - AUTHOR RESPONSE

Reviewer: 1
Dr. Venkatesh Chandrasekaran, Jawaharlal Institute of Postgraduate Medical Education and Research
Comments to the Author:
An interesting study involving a large number of children to assess whether overcrowding is associated with unfavorable indoor living environment, respiratory and allergic disorders and poor general health. This study also attempts to assess whether children with asthma or those from economically underprivileged areas are more exposed or affected by overcrowding. Thank you very much Dr. Chandrasekaran for taking the time to review our manuscript and for your valuable feedback.

The outcome measures have been collected as yes/no answers from the respondents making the measure very subjective

Thank you for this comment. However, this is not entirely accurate. The survey questions were multiple choice and had more answer options than simply binary yes/no answers. Example: general health was answered in the survey with "very good", "good", "moderate", "bad", "very bad". As stated in the Methods section (page 10 under "living environment and health") for the purpose of data analysis, in this case a logistic regression model, we have combined survey answers and created new variables to obtain binary variables. Example: we created a new variable "good general health" if people answered, "very good" or "good". See the example of the definition of asthma as additional example. This might have not been very clear from the description, so we have tried to reformulate our wordings to avoid misunderstandings in the Methods section, subheading Indoor living environment and health, page 10. Unfortunately, word limits restrict the detailed description of all the respective original survey questions. Nevertheless, the survey questions remain subjective, which is from the authors' point of view even desirable as we would like to capture the individuals' personal perception on home, health and wellbeing outcomes.

The authors have used two different sets of criteria to measure overcrowding- a stringent criterion (norm 3) in which if more than one person sharing the same bedroom other than their partner and a liberal criterion (norm 2) in which >2 individuals sharing a bedroom is considered overcrowding. In this context, it would have been interesting if the authors had also collected information about the size of the bedroom, as well as cultural context facilitating sharing of the same space. For instance, it is common among Indian Children to share bed with their parents in most households until they become adolescents.

This is a very interesting reflection, thank you for sharing. It is certainly very valuable to look at our findings from a very different context as well. We would just like to mention here, that it is not uncommon in Sweden as well, for very young children to sleep in the parental bedroom. We also acknowledge that some of the outcomes in our study, in particular general health, may be more relevant for the children at 12 years and older than for younger children, as they increasingly need and value their privacy while transitioning into adolescence. Yet as our sub-analyses show, the main findings barely differ from the total children in our study. We agree it would have also been interesting and a strength to consider the size of the bedroom and cultural context, but unfortunately this was not within the scope of our study as the information was not available through the questionnaire or register data as administered by the Swedish Public Health Agency. However, we would like to mention that most of the children in our study were of Swedish origin (75%), as displayed in Table 1, which gives an idea of the cultural context in this study.

Interestingly the authors have used a term "criterium" which I understand it to be criterion.

Thank you for pointing this out; we have now changed the wording to "criterion", page 5 in the introduction.

No formal sample size calculation has been mentioned for this study. The study has reportedly used multi-cluster sampling to recruit children in the study with each county recruiting about 600 children with 200 in each of the 3 age group categories, 6-10 months, 4 years and 12 years. It is not clear how the rest of the sample was recruited considering

there were only three counties mentioned in the results- Stockholm, Vastra Gotaland and Skane. It is also not clear what was the rationale for selecting these different age groups.
The authors would like to highlight at this point that the sample size calculation, the selection of study participants and the corresponding in- and exclusion criteria were not formally part of this study, hence the absence of a study flow diagram. As described in the Methods, the survey was conducted and administered by the Swedish Public Health Agency (FHM) in collaboration with Statistics Sweden, independent of this study. The age groups were chosen by FHM, and are meant to represent infants, young children and school children. Given the strict word limit, we have tried to keep the description of the survey operationalisation as short as possible, since this was also not part of this study. Since the FHM encourages use of their data for research purposes, we have been privileged to be able to use the data from this survey for our study. We have received a finished dataset to do our analyses. To clarify this, we have revised our wording in the Method section and hope that it is now clear that the survey administration was outside the scope of this study and that the sample was recruited apart from the metropolitan areas (Methods section, subheading Study population, pages 8-9). The more precise reason why we chose those 3 counties is further elaborated on in a comment below (second last).

The authors considered a diagnosis of asthma in the child if the parents have been told by the doctors about it, or if the child is on asthma medications or prevalence of breathing difficulty in the past 12 months. In this context, it is important to note that some of the children would have outgrown the symptoms, might not be on medications currently and might not be having any symptoms in the past 12 months making the measurement inaccurate.
For this study we have followed the definition as used in the Swedish National Environmental Health report to define a child with asthma. We believe that this definition is a strong one and will capture all the children that we would like to capture, as we would want also to focus on children that experience at least some asthma symptoms, and less so on children that may have an asthma diagnosis on paper but do feel completely free of symptoms and use no medication. However, we have doublechecked in our dataset how many children we would “miss” with our “two out of three” rule as a definition. There are 3,668 children that have doctor’s diagnosis of asthma but 4,087 children that have asthma according to our “two-out-of-three” rule. This means that we do not seem to miss any children but rather include a few more children that experience asthma symptoms but lack a formal doctor’s diagnosis, which is in line with our research aim. We hope that this addresses your concerns sufficiently.

Parental perception of good general health of their children might vary considerably. Instead, the authors could have used a surrogate like the absence of poor weight gain and or poor appetite, frequent infections or need for frequent hospitalizations due to ill health. Similarly parental perception of poor air quality might also not be reliable. Instead, the authors could have used air quality index values of the neighborhoods acquired from nearest weather monitoring stations. Similarly, perception of too much cold indoors in the winter and too much heat in the summers could be very subjective
Thank you for these valuable suggestions. We agree that it would have been very interesting to complement our outcome variables with indicators of appetite, infections etc. However,

due to the predefined questions, as selected and defined by the Swedish Public Health agency when designing their survey, such indicators were not available for analysis. We were limited to the information as captured by the survey. Nevertheless, we do not believe that subjectivity is a major issue in this study, as already mentioned above, our aim was to actually capture the subjective perception of the parents respectively the child to answer our research question. So, we do in fact value the subjectivity in this case as a strength.

A STROBE compliant study flow diagram depicting patient flow into the study is missing and the authors have stated it is not applicable in the STROBE checklist, whereas it is very much needed to understand how many were found to be eligible for participation, how many met the study inclusion criteria, how many were excluded and for what reasons and how many were analysed finally.
The authors would like to refer to the point above concerning the sample size calculation. Meaning that the sample size calculation, the selection of study participants and the corresponding in- and exclusion criteria were not formally part of this study, and hence the absence of a study flow diagram. We do, however, provide information on total and age specific target population size, sample size, number of responders and response rates (%) as specified by the Swedish Public Health Agency in Table S1 which provides necessary information on participation rates etc. for the present study.

In table 1 the number of children belonging to different counties do not count up to 47,033.
Thank you for pointing this out, this is correct and meant to be this way. The three mentioned counties do not represent the full study population. Sweden has in total twenty-one counties and each county is represented in the sample. We are just highlighting the counties of Stockholm, Skåne and Västra Götaland in subanalyses because those counties represent the main metropolitan areas in Sweden; areas which are suspected to be most at risk for crowded housing due to high urban density. A bit more than half of the children in our sample are living in those three counties (N=25,100).

Reference number 8 appears to be incomplete.
This is accurate and we have now completed the reference. We have also completed a few others, reference 4, 6, 20, 33, 39, 40. There seems to have been an issue with the citation generation for some reports. Thank you.

Reviewer: 2
Dr. Ren Chen, Anhui Medical University
Comments to the Author:
Thank you for the invitation to review this manuscript. This article examines the relationship between indoor living environments, crowded housing, and childhood allergies and respiratory diseases in Sweden, providing insights for other scholars studying the environment and children's health. I have some concerns about certain parts of the manuscript and hope that the author will address these issues after careful consideration.
Thank you very much Dr. Chen for taking the time to review our manuscript and for your valuable feedback.

1. In this study, the health outcomes for children included asthma, airway issues, breathing

difficulties, mold and dust mite allergies, pollen allergies, furry pet allergies, and overall good health. Furthermore, the entire study primarily focused on children's allergies and respiratory diseases. However, the current title is "......and children's health......," which may not align closely with the theme and feels somewhat broad. It is recommended that the author consider revising the title to make it more rigorous and targeted.
Thank you for this great suggestion. We agree and have changed the title accordingly to "*Crowded housing, indoor environment and children's respiratory, allergic and general health in Sweden: a cross-sectional study*".

2. In the background section, the author describes the impact of overcrowded housing and poor indoor environments on children's health, as well as previous research findings. However, there is limited discussion of children's allergies and respiratory diseases themselves. Even when mentioned, they are often discussed in conjunction with poor living conditions. Therefore, it is recommended that this aspect be further elaborated upon.
This is a valid comment, and we have added now some more background information on children's allergies and respiratory diseases, whilst considering the limits of the overall word count (Introduction paragraph 5, pages 6-7).

3. Adding information about the current situation of respiratory diseases and allergies among Swedish children to the research background would make the article more complete.
The authors agree and have added some national statistics on respiratory diseases and allergies in the Introduction paragraph 5, pages 6-7).

4. The review of previous studies could be more detailed to present a clearer picture for the readers. For example, "A study of XX participants (here you can clearly state what the sample is) using XX method found XX."
Yes, we have now reformulated some of the sentences in the Introduction to provide a greater level of detail about the methods of existing studies (Introduction paragraph 5-6, page 7-8. We hope that this provides now a better understanding of the current literature.

5. In the subgroup analysis of the statistical analysis, what is your definition of the three major metropolitan counties of Stockholm, Västra Götalands, and Skåne? Or what is the basis for your selection? Please provide additional information to clarify this point.
Thank you for these questions. Sweden has in total twenty-one counties, and each county is represented in the sample. We are highlighting the counties of Stockholm, Skåne and Västra Götaland in subanalyses because those counties represent the metropolitan areas of the country – including the three largest cities of Sweden; areas which are suspected to be most at risk for crowded housing due to high urban density. These three counties are also the only counties that can be considered as metropolitan. We have tried to make the basis of selection clearer in the manuscript now (Methods section, Study population, page 9).

6. It is recommended that the description of population characteristics be more detailed.
Thank you for the suggestion. We believe that our tables do already contain a high level of detail, but we have elaborated now still the in-text descriptive part of the population

characteristics, whilst taking the overall word limits into account. We hope that this now provides a sufficient level of detail (Results section, Population characteristics, page 13).

7. When describing the results, subjective inferences should not be made. The results section should only objectively present your findings.
We agree with the reviewer that it is not appropriate to make causal inferences in the results sections and believe that we now don't make any subjective inferences in the Results section. We have scrutinized the text to make sure that we only indicate where we see associations and don't imply causality. Thank you for pointing this out.

8. Specific, actionable recommendations should be proposed in response to the identified issues and challenges.
The authors agree that this is a very important point and that this may have been insufficiently addressed in the first submission. We have added a subsection at the end of the Discussion, page 21, with more precise recommendations and hope this adds to the strength and use of this study. Thank you for this valuable suggestion.

9. Carefully check the correspondence between text descriptions, charts, and textual data.
Thank you for the recommendation. We have now doublechecked the alignment between the different text and graphical parts of the manuscript.

VERSION 2 - REVIEW

Reviewer 1

Name Chandrasekaran, Venkatesh

Affiliation Jawaharlal Institute of Postgraduate Medical Education and Research, Department of Pediatrics

Date 03-Sep-2025

COI

The authors have addressed all my queries in a satisfactory manner
